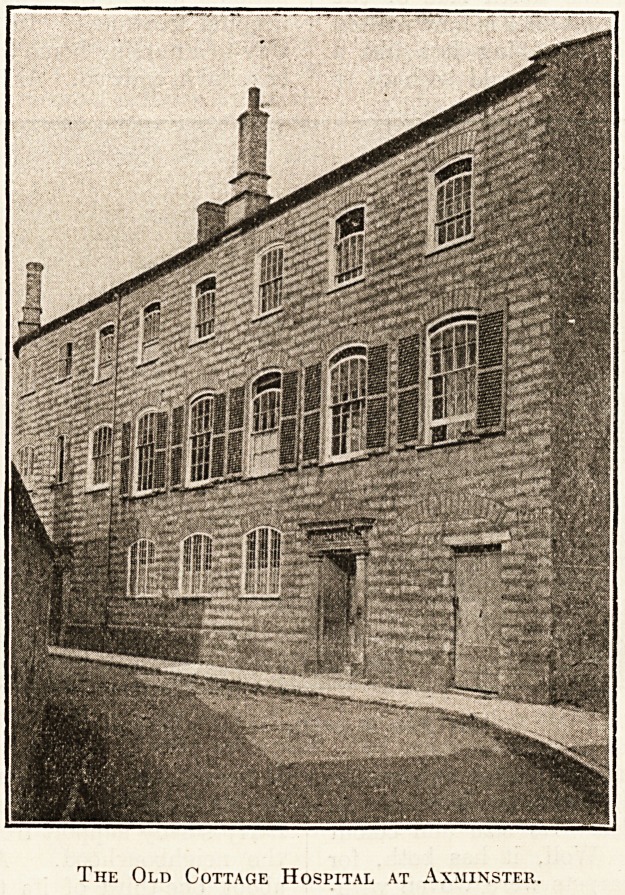# The Axminster Cottage Hospital

**Published:** 1911-11-11

**Authors:** 


### THE AXMINSTER COTTAGE HOSPITAL.

### A Transition to the New from the Old.

There is sufficient material to form an interesting historical sketch of this little institution which was started on its present curious site in 1886 by Miss Emily Conybeare. It is to move next year to the modern buildings that are now almost finished in another part of the town. As readers of The Hospital will remember, the foundation-stone of the new buildings was laid a few weeks ago.

The first question that a stranger might ask on entering Axminster would probably be as to where in so small a community its famous carpets are made. The industry has departed, but the factory in which it was carried on still remains, a grey three-storeyed building on the north side of the parish church, but over its front door is now written "The Cottage Hospital." Anything less like a cottage or less like a hospital it would be hard to imagine. The building, as the photograph on the next page emphasises, looks more like an old palazzo than anything else. Its size and colour betoken dignity and age. Well, it has both, for formerly the Axminster carpets were woven within. The interior is no less interesting. It is as little like a factory indoors as like a cottage hospital without. A series of spacious rooms open out of a long passage at the back, the windows of which give a view of cottage roofs and a deserted garden. The walls dividing some of the rooms from each other and apparently the house from its neighbour are extremely thin, being lath-and-plaster partitions, and showing that this extensive habitation is larger even than the hospital itself. The Conservative Club and the armoury of the Territorials have absorbed its westernmost division. At one end of the long passage alluded to is an incredibly abrupt and narrow stair by which an agile person can climb to the first floor, but up which it must be extremely difficult to carry an invalid or anyone unable to walk. On this floor there is another passage and a similar set of rooms, two of which are wards; the third is used for operations, the narrow table in the centre betraying its purpose. The simple boards and plain walls are very different from the tiled palaces in which London surgeons work, but we have very little doubt that the work has not suffered from this simplicity.

Like many another institution, this hospital has grown up from very small beginnings, the starting-point in this case being the desire of the foundress to provide a convalescent home for local patients who in those days were sent to Exeter for hospital treatment. In process of time this gave way to a nursing home which was really the cottage hospital in embryo. As in similar cases, an increase in the number of patients and of operations has been recorded, and the hospital has managed to secure a fairly steady support from the well-to-do classes of the neighbourhood. Annual subscriptions form about one-third of its total income and approximately half of the yearly amount subscribed, and a balance at the bank has enabled it on the whole to keep clear of debt. The farmers are not strong supporters, but there are signs that the cottagers have a genuine desire to help towards the maintenance of the hospital, to which they are very glad to go in times of illness or accident. One reason perhaps why the hospital has kept clear of debt has been the opinion that it would be useless to tinker with a building which offered such inherent difficulties for the institutional treatment of disease. The feeling was that enough money must be subscribed to make rebuilding possible, and the only question was when had sufficient been obtained to warrant rebuilding.

The New Hospital as it will be.

Two legacies were found in 1911 by interest to have reached approximately £800, and this amount was set aside to form the nucleus of the building fund. The death of King Edward suggested to the Committee the idea of associating his name with the scheme for a new hospital, and in January 1911 an appeal was issued. This appeal was a simple summary of the disadvantages of the old, that is, the present building. It reminded the hospital's supporters that the stairs made all access, whether by patients or the staff, to the upper floors exceedingly difficult; that the building had never been designed for hospital purposes and could not be adapted to them; and finally, that the nearness of the parish church and the thinness of the partition walls dividing the hospital from the rest of the building alike made the bells and the movements of anyone next door distressingly noisy to the patients. This was a direct and strong case, and has been recognised as such, since £1,100 has been subscribed in the months since the date of its issue. The Coronation was honoured by giving subscriptions to the hospital, and the smallness of many of the sums which one notices in the list of those who gave to the building fund proves how wide a support the proposal has gained among the poorer classes. This is all the more gratifying as it should secure the maintenance of the new building by paving the way for numerous small annual subscribers, by which alone a hospital's solvency can be satisfactorily secured.

The extraordinary progress of hospital construction and equipment in the past half-century could hardly be traced more clearly than by the comparison of the old buildings with those now nearing completion. The design was thrown open to competition and the successful plans were those of Mr. T. Leslie Moore, A.R.I.B.A. It is, we believe, the second cottage hospital which he has built, and our first illustration will give some idea of the way in which he has treated the site and of the attractiveness with which he has invested the appearance of the buildings, which are faced with grey rough-cast and roofed with red tiles.

We shall hope to publish the plans at the date of the opening, and till then confine ourselves to the simplest description. On either side of the entrance hall are respectively the board-room and the matron's sitting-room; overhead are the probationers' and matron's bedrooms. One passes on to the first of the two wards by way of a room which is to be reserved for private patients. At the far end of the first ward is a small room, called by Mr. Leslie Moore the duty room, which lies immediately between it and the second ward, which rounds off the garden at this point. The duty room by means of two small windows commands a complete view of both wards, and thus simplifies control and administration. Just beyond it is the kitchen, below which is a basement washhouse, and above the servants' bedroom: thus the staff for all purposes can remain indoors. A modern theatre has been built and cross-ventilation is secured throughout. This very rough description is enough to show that the plans are worthy of some study; an opportunity for that we shall hope to give at a later date.

The Old Cottage Hospital at Axminster.

Some £200 more are needed to pay the cost of building, which is rather more than £2,000. Then £250 will be required for furnishing and equipment. That supplied, Axminster will have a cottage hospital of which it will be proud, and for the maintenance of which the signs are not lacking.

No better or more interesting appeal could be found than the simple comparison of the old and new hospital, the gradual development of which is a curious epitome of hospital evolution which it would be well worth while summarising in the next annual report. The interest taken by Mr. Hoel Hacon, the hon. secretary, who has worked hard at pressing the claims of the new building, is that which the voluntary system has nobly evoked in many others, and the local interest in this hospital is aptly shown by the long connection with it of Mr. R. Cornish, J.P., who resigned the secretaryship eighteen months ago, after fourteen years of untiring labour, only to become the hospital's President.

The new and the old hospital for a short while will remain side by side, public monuments of cottage hospital progress, till the old passes to yet another purpose, with little reminder perhaps of its curious history as a famous factory turned hospital, now that its patients, like its looms and carpets, have removed elsewhere.

## Figures and Tables

**Figure f1:**
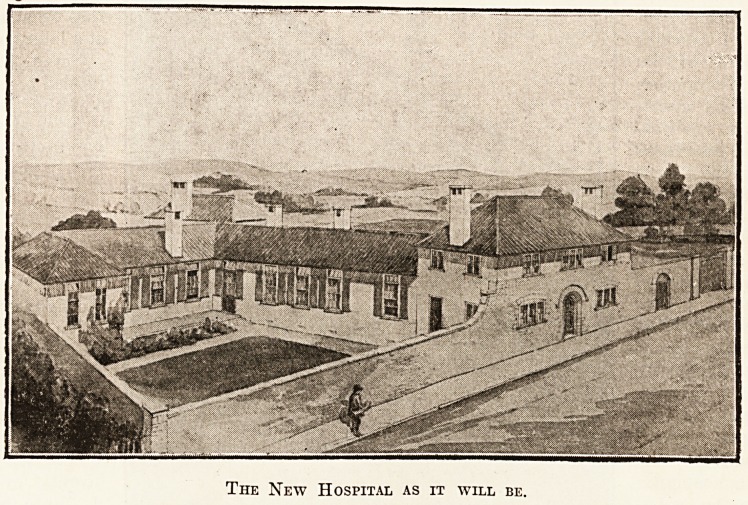


**Figure f2:**